# The Prevention of Cholera

**Published:** 1910-09-24

**Authors:** 


### Public Health and Hygiene.

### THE PREVENTION OF CHOLERA.

Not unnaturally, when cholera reaches a port so near to our shores as Rotterdam, apprehension arises as to the danger of the disease invading the British Isles. The number of people arriving in this country from the infected ports is very considerable, and the possibility of cholera being introduced by persons incubating the disease cannot be overlooked. Fortunately the incubative period of cholera is so short, varying from a few hours to three or four days, that the majority of cases contracting the disease in a foreign port would show symptoms before their arrival in England. Still, the fact cannot be overlooked that it is possible that persons might arrive in London or Hull apparently in perfect health, who, a few hours after their arrival, might be seized with cholera of which they presented no evidence at the port of disembarking. To meet this contingency, the practice has been adopted of taking the names and addresses of all persons arriving from infected ports, and these are forwarded by the port medical officers to the medical officers of health of the respective districts to which such persons are bound. Upon receipt of these notifications made by the port medical officer, the medical officer of health makes inquiry at the address given as to the health of the person, or persons, of whose arrival he has been notified, and arranges to be made acquainted with any symptoms of suspicious illness should they unfortunately arise. By this means it is anticipated that he would get the earliest possible knowledge of any imported case and so be in a position to stop the further spread of the disease.

### The Risk in England.

The medical officers of health of considerable towns are constantly receiving notifications of such cases, and it is part of their routine work to make, or arrange for, the necessary inquiries. It not infrequently happens that persons arriving in a home port, either intentionally or through inadvertence, give a wrong address as their destination, and in this lies one of the weak links in the chain of protection which has been devised against the invasion of the disease. Should any of these persons develop the disease an outbreak of considerable dimensions might occur before it came to official knowledge. The risk of any considerable epidemic of cholera in this country is admittedly very small. Like typhoid fever, its epidemic prevalence is dependent chiefly upon an infected water supply. The water supplies of most of the larger centres of population in Britain may be considered exempt from any risk of serious contamination. London, dependent as it is for its water supply upon the large watersheds of the Thames and Lea, might be considered to be exposed to extensive risks, but the care with which the water is filtered before being distributed for public consumption confers upon it a practical immunity from danger, as is shown in the low incidence of typhoid fever to which it is subject.

Secure as the country seems to be against cholera invasion, however, it must not be forgotten that from time to time outbreaks of the disease occur. Should it gain a footing, the risks of personal infection as in typhoid fever are not negligible.

The immense success which has attended the perfecting of our public water supplies in checking the spread of water-borne diseases has tended to blind us to the minor ways in which these diseases are diffused. Outbreaks of typhoid fever of considerable dimensions have repeatedly been recorded which have no relation to a defective water supply, and protective schemes against such a comparatively remote contingency as cholera are not complete in closing merely that channel of spread.

Should cholera be introduced into this country what steps would be taken to limit its spread?

These may be classified under the following heads: (1) Facilities for diagnosis; (2) notification; (3) isolation; (4) disinfection; (5) observation of contacts and suspicious cases.

### Facilities for Diagnosis.

The aid to diagnosis which bacteriology affords would be placed at the disposal of medical practitioners by all the larger sanitary authorities and by the counties where no such provision has been made by the local authorities. In addition to this the services of medical men experienced in the disease would doubtless be secured by the local authorities in large towns. The London County Council made such provision both in the case of plague and smallpox, and would doubtless do so were cholera to be introduced.

It is only when provision of this kind is made that the full value of notification is obtained. The general practitioner hesitates to notify doubtful cases of a disease with which he is necessarily unfamiliar, and it is immensely to the advantage of the health authority to render him every assistance in furnishing them with information so material to the public welfare.

### Isolation of the Patients.

In the provision of infectious diseases hospitals this country is fortunately situated. Consideration of the patient, however, would in many cases preclude his removal to them. His condition will not permit of removal to any great distance, and in many cases not at all. In a report made in 1885, relative to cholera in London, Sir Shirley Murphy pointed out that "persons attacked will not be equally distributed over the whole metropolis, but will be concentrated in some part or parts too distant from many of the hospitals which the managers would have provided for these hospitals to be of any service to the infected district."

It would therefore in many instances be necessary to make the patient's house the hospital, and to remove the contacts, or supplementary hospitals would be extemporised out of dwelling houses in the infected districts.

Observation of Contacts.

A most important provision in the preventive measures taken would be that for observation of the contacts. Those which are removed would have to be taken to suitable stations and those kept under supervision until the incubative period had elapsed. The arrangements for carrying out the different items of such a scheme would necessarily vary with the nature of the district in which they were rendered necessary.

In London, the County Council would doubtless assume a large measure of responsibility in the protection of the metropolis. Diagnostic facilities, both consultative and bacteriological, would doubtless be provided by the County Council, as in the case of plague. Visitation and investigation of suspected cases in their homes and the housing and supervision of contacts and suspects could only economically be effected by the authority having administrative jurisdiction over London as a whole. So far as was practicable the Asylums Board hospitals would receive the patients for isolation, but supplementary hospitals would have to be provided and with these medical and nursing attendance on the sick. Notification of cases would be made to the borough medical officers of health, who would investigate them, trace the movements of the patients, and follow up the contacts, arrange for the removal or isolation of the sick, and the disinfection of the premises.

There is, fortunately however, little ground for anticipating that these or similar arrangements may be necessary. Nevertheless, to be prepared is to avert disaster in so momentous though improbable an issue.

